# Identification of *Rickettsia* spp. and *Babesia conradae* in *Dermacentor* spp. Collected from Dogs and Cats Across the United States

**DOI:** 10.1089/vbz.2021.0047

**Published:** 2021-12-16

**Authors:** Kathryn T. Duncan, Amber Grant, Britny Johnson, Kellee D. Sundstrom, Meriam N. Saleh, Susan E. Little

**Affiliations:** ^1^ Department of Veterinary Pathobiology, College of Veterinary Medicine, Oklahoma State University, Stillwater, Oklahoma, USA.; ^2^ Rickettsial Zoonoses Branch, Division of Vector-Borne Disease, National Center for Emerging and Zoonotic Infectious Diseases, Centers for Disease Control and Prevention, Atlanta, Georgia, USA.; ^3^ Department of Veterinary Pathobiology, College of Veterinary Medicine and Biomedical Sciences, Texas A&M University, College Station, Texas, USA.

**Keywords:** *Dermacentor variabilis*, *Dermacentor albipictus*, tick, pet, *Rickettsia*, *Babesia*

## Abstract

In the United States, *Dermacentor variabilis* and *Dermacentor andersoni* are considered key vectors for *Rickettsia rickettsii*, the causative agent of Rocky Mountain spotted fever. Through regional surveillance, a wide diversity of *Rickettsia* spp. have been documented in *D. variabilis*, and *Dermacentor* spp. has been suggested as potential vectors for various other pathogens, including *Babesia* spp. and *Ehrlichia canis*. To better define the prevalence and diversity of pathogens in *Dermacentor* spp. across the United States, 848 ticks collected from dogs and cats in 44/50 states in 2018–2019 were tested by PCR for *Rickettsia* spp.-specific 17 kDa and ompA gene fragments; a subset of *Dermacentor* spp. was also tested with PCR, targeting fragments of the 18S and large subunit region rRNA genes of *Babesia* spp. and 16S rRNA genes of *E. canis*. *Rickettsia* spp. was identified in 12.5% (106/848) of ticks. Species detected include *Rickettsia montanensis* (*n* = 64 ticks), *Rickettsia bellii* (*n* = 15 ticks), *Rickettsia rhipicephali* (*n* = 13 ticks), *Rickettsia peacockii* (*n* = 8 ticks), *Rickettsia amblyommatis* (*n* = 3 ticks), *Rickettsia cooleyi* (*n* = 1 tick), and unclassified *Rickettsia* spp. (*n* = 2 ticks). Ticks with *R. montanensis* and *R. bellii* were submitted from every U.S. region; *R. rhipicephali* was predominantly detected in ticks from the southern half of the United States, and all *R. peacockii*-positive ticks were *D. andersoni* that originated from the Rocky Mountain states. *Ehrlichia canis* was not detected in any *Dermacentor* spp., and *Babesia conradae* was detected in two *Dermacentor albipictus*. Because most ticks had fed on dogs or cats before submission, these findings do not implicate a given *Dermacentor* sp. as a primary vector of these agents, but in regard to *Rickettsia* spp., the data do support other published work showing *D. variabilis* harbors a diversity of *Rickettsia* species with unknown implications for animal and human health.

## Introduction

Ticks transmit more vector-borne disease agents in the United States than any other arthropod (Parola et al. 2005). Tick-borne rickettsial pathogens are widely distributed across this region and pose a significant health risk to both humans and animals as nontreated, and some treated, cases may end in death (CDC 2004, Parola et al. 2013, Levin et al. 2014, Biggs et al. 2016, Jiang et al. 2021). In the United States, Rocky Mountain spotted fever (RMSF) caused by *Rickettsia rickettsii* is historically the primary agent of severe rickettsiosis in humans and dogs (Biggs et al. 2016). In humans, disease is characterized by a petechial rash, but infected individuals may initially present with fever, headache, malaise, and myalgia. RMSF in dogs can manifest as fever, lethargy, decreased appetite, and tremors with an occasional maculopapular rash on areas of exposed skin (Levin et al. 2014, Biggs et al. 2016). Classically, *Dermacentor variabilis* in the eastern United States and *Dermacentor andersoni* in the western United States were considered the primary vectors for *R. rickettsii* in the region. However, *Rhipicephalus sanguineus sensu* lato (s.l.) has been identified as a competent vector in the southwestern United States and *Amblyomma americanum* has also been implicated as a secondary vector (Demma et al. 2005, Breitschwerdt et al. 2011, Biggs et al. 2016, Levin et al. 2017, Saleh et al. 2021).

Reports and concern for spotted fever group *Rickettsia* (SFGR) other than *R. rickettsii* as a significant cause of human disease have grown over the last two decades (Delisle et al. 2016, CDC 2018). For instance, *R. philipii* (*Rickettsia* 364D) transmitted by *Dermacentor occidentalis* in California may cause fever, headache, or a maculopapular rash, while *Rickettsia parkeri*, transmitted by *Amblyomma maculatum* across its southeastern range, can cause similar signs in humans (Parola et al. 2013, Paddock et al. 2018, Yoshimizu and Billeter 2018). In addition, a human case report describes rickettsiosis following a *D. variabilis* bite caused by *R. montanensis*, and recently, a novel *Rickettsia* sp. was identified as the cause of fever, lethargy, and thrombocytopenia in three dogs from south central U.S. states (McQuiston et al. 2012, Wilson et al. 2020). As many as 60% of dogs in disease-endemic areas may have evidence of exposure to SFGR (Levin et al. 2014). However, because of the cross-reactivity of various SFGR on routinely used *Rickettsia* indirect fluorescent antibody (IFA) assays, novel pathogens such as the one recently isolated from dogs, as well as other SFGR, likely go unrecognized (Parola et al. 2005, Wilson et al. 2020). Investigators in several U.S. regions have surveyed *D. variabilis* for *Rickettsia* spp. and demonstrated this tick carries a variety of SFGR, and, of particular importance, that *R. rickettsii* is rarely detected in *D. variabilis*, which is significant, given this tick is currently considered a primary vector for this pathogen (Table 1).

**Table 1.** Representative Reports of *Rickettsia* spp. detected in *Dermacentor variabilis* in the United States, 2000–Present

U.S. region and state	Source(s) of ticks	Rickettsia spp.	Prevalence (%)	References
West				
California	Humans, environment	*R. bellii*	88.2–88.4	Hecht et al. (2019); Osborne et al. (2020)
	Humans	*R. montanensis*	2.2	Stromdahl et al. (2011)
	Environment	*R. rhipicephali*	40.0	Wikswo et al. (2008)
Washington	Environment	*R. bellii*	6.8	Hecht et al. (2019)
	Environment	*R. montanensis*	1.9	Hecht et al. (2019)
	Environment	*R. rhipicephali*	1.0	Hecht et al. (2019)
Midwest				
Kansas	Humans, environment	*R. montanensis*	10.5	St. John et al. (2016); Hecht et al. (2019)
Minnesota	Humans, environment	*R. montanensis*	1.6–31.9	Stromdahl et al. (2001); St. John et al. (2016); Hecht et al. (2019)
Missouri	Environment	*R. amblyommatis*	NR	Santanello et al. (2018)
	Environment	*R. montanensis*	NR	Santanello et al. (2018)
Nebraska	Environment	*R. amblyommatis*	0.5	Luedtke et al. (2020)
	Environment	*R. bellii*	0.4	Luedtke et al. (2020)
	Environment	*R. montanensis*	3.1	Luedtke et al. (2020)
North Dakota	Environment	*R. montanensis*	4.3	Hecht et al. (2019)
	Environment	*R. rhipicephali*	4.3	Hecht et al. (2019)
Wisconsin	Humans	*R. montanensis*	0.3–0.6	Stromdahl et al. (2001); St. John et al. (2016)
South				
Arkansas	Dogs	*R. montanensis*	2.2	Trout Fryxell et al. (2015)
Georgia	Environment	*R. bellii*	2.8	Hecht et al. (2019)
	Domestic animals	*R. felis*	0.6	Stanley and Rhodes (2021)
	Environment, domestic animals	*R. montanensis*	4.2–14.7	Hecht et al. (2019); Stanley and Rhodes (2021)
Kentucky	Humans, environment, domestic and wild animals	*R. amblyommatis* ^a^	0.8–1.1	Fritzen et al. (2011); Hecht et al. (2019)
	Environment	*R. bellii*	8.9	Hecht et al. (2019)
	Humans, environment, domestic and wild animals	*R. montanensis* ^b^	0.3–4.5	Fritzen et al. (2011); Pagac et al. (2014); St. John et al. (2016); Hecht et al. (2019)
	Humans, environment, domestic and wild animals	*R. parkeri* ^c^	0.6–2.4	Fritzen et al. (2011); Hecht et al. (2019)
	Environment	*R. rickettsii* ^c^	0.8	Hecht et al. (2019)
Mississippi	Environment	*R. bellii*	2.0	Hecht et al. (2019)
North Carolina	Environment	*R. amblyommatis*	29.3	Kakumanu et al. (2018)
	Environment	*R. bellii*	1.9	Kakumanu et al. (2018)
	Environment	*R. canadensis*	1.3	Kakumanu et al. (2018)
	Environment	*R. conorii* like	3.6	Kakumanu et al. (2018)
	Environment	*R. massiliae*	3.9	Kakumanu et al. (2018)
	Environment	*R. montanensis*	7.7	Kakumanu et al. (2018)
	Environment	*R. parkeri* ^c^	7.9	Kakumanu et al. (2018)
	Environment	*R. rhipicephali*	0.4	Kakumanu et al. (2018)
	Environment	*R. rickettsii* ^c^	0.9	Kakumanu et al. (2018)
	Environment	*R. typhi*	1.3	Kakumanu et al. (2018)
Oklahoma	Humans	*R. montanensis*	0.3	Stromdahl et al. (2001)
Tennessee	Humans, environment, wild and domestic animals	*R. amblyommatis* ^a^	2.5	Moncayo et al. (2010)
	Humans, environment, wild and domestic animals	*R. montanensis* ^b^	4.0–9.5	Moncayo et al. (2010); Pagac et al. (2014); St. John et al. (2016)
Virginia	Environment	*R. amblyommatis* ^a^	0.04–4.2	Henning et al. (2014); Hecht et al. (2019)
	Environment	*R. bellii*	1.9	Hecht et al. (2019)
	Humans, environment	*R. montanensis*	0.2–1.4	Stromdahl et al. (2001); Henning et al. (2014); St. John et al. (2016); Hecht et al. (2019)
	Environment	*R. parkeri* ^c^	0.7	Henning et al. (2014)
West Virginia	Humans	*R. montanensis*	NR	St. John et al. (2016)
Northeast				
Connecticut	Humans	*R. montanensis*	NR	St. John et al. (2016)
Maryland	Humans, environment	*R. montanensis*	0.5–3.8	Ammerman et al. (2004); St. John et al. (2016)
	Humans	*R. rickettsii* ^c^	0.02	Stromdahl et al. (2011)
New Jersey	Humans, environment	*R. montanensis*	0.4–1.3	Stromdahl et al. (2001); St. John et al. (2016); Occi et al. (2020)
New York	Environment	*R. amblyommatis*	8.3	Hecht et al. (2019)
	Environment	*R. montanensis*	8.3	Hecht et al. (2019)
Pennsylvania	Environment	*R. bellii*	1.8	Hecht et al. (2019)
	Humans, environment	*R. montanensis*	0.2–3.6	St. John et al. 2016; Hecht et al. (2019)
Rhode Island	Humans	*R. montanensis*	NR	St. John et al. (2016)

Detection of a given pathogen, alone, does not confirm vector competence.

^a^ Reported as *Rickettsia amblyommii* in one or more reference.

^b^ Reported as *Rickettsia montana* in one or more reference.

^c^ Commonly associated with human disease in the United States.

NR, not reported. Prevalence, or data to calculate prevalence, was not included in the reference.

In addition to SFGR, *Dermacentor* spp. has been implicated as vectors for other pathogens in the United States. Experimentally, *D. variabilis* can transmit *Ehrlichia canis* to dogs, causing ehrlichiosis, a potentially life-threatening illness with some dogs showing no sign of infection, while others display evidence of fever, lethargy, anorexia, myalgia, lymphadenopathy, bleeding tendencies, and neurologic abnormalities (Johnson et al. 1998, Little 2010). Also, a rapidly emerging and medically relevant pathogen in the western United States and Canada, *Babesia duncani*, has caused serious disease in immunocompromised humans, and preliminary research suggests *Dermacentor albipictus* as one of the tick vectors (Swei et al. 2019, Yang et al. 2021). The winter tick, *D. albipictus*, is occasionally found on dogs and cats, although it is maintained in nature by wild ungulates (Duncan et al. 2020).

Various *Dermacentor* spp. is encountered questing in the environment across its range and is commonly collected from dogs and cats (Thomas et al. 2016, Little et al. 2018, Lehane et al. 2020, Saleh et al. 2021). In a survey of ticks infesting dogs and cats across the United States, *D. variabilis* was the tick species most commonly infesting dogs (Saleh et al. 2019). Recently, *D. variabilis* was shown to be the predominant *Dermacentor* spp. infesting pets in the United States, including in the Rocky Mountain states, where this tick was historically considered rare or absent (Duncan et al. 2021). With the potential to spread pathogenic agents coupled with their changing geographic distribution, *Dermacentor* spp. surveillance and pathogen screening remain warranted (Minigan et al. 2018, Sonenshine 2018, Lehane et al. 2020, Duncan et al. 2021). In addition, pets may be considered sentinels for both ticks and tick-borne pathogens, and those with evidence of disease may indicate a risk to other members of the household (Paddock et al. 2002, Levin et al. 2014). Hence, the objective of this study is to describe the diversity and geographic distribution of potential pathogenic organisms in *Dermacentor* species ticks removed from dogs and cats across the United States.

## Materials and Methods

### Tick samples

*Dermacentor* spp. (*n* = 848; 544 females, 297 males, and 7 nymphs) from 471 dogs (*n* = 770 ticks) and 56 cats (*n* = 78 ticks) from 44/50 states used in this study was obtained and identified through an ongoing national survey of ticks on dogs and cats as previously described (Saleh et al. 2019, Duncan et al. 2021). Ticks were collected in private veterinary practices during the course of normal physical examinations; institutional review board approval was not required. Ticks were selected for *Rickettsia* spp. testing with a goal of achieving extensive geographic and *Dermacentor* species representation. Since *D. variabilis* was identified in all 44 states with *Dermacentor* spp. submitted, *D. variabilis* specimens (*n* = 827) were selected for use to reflect a broad geographic distribution across the four U.S. regions (Northeast, South, Midwest, and West) (Blagburn et al. 1996, Duncan et al. 2021). *D. andersoni* (*n* = 12) and *D. albipictus* (*n* = 9) were included to diversify the species tested, even though low numbers were submitted. To determine if less commonly identified pathogens were present, a subsample of ticks (*n* = 150) from the southern United States where canine ehrlichiosis is known to occur was tested for *E. canis*, and a subset of ticks (*n* = 189) primarily from the southern United States and from along the Atlantic and Pacific Coasts where babesiosis has been reported or suspected to occur was tested for *Babesia* species (Beall et al. 2012, Birkenheuer et al. 2020, Little et al. 2021).

### Molecular assays

Each evaluated tick was dissected dorsoventrally and all internal contents collected for nucleic acid extraction, which was performed using a commercial blood kit with no modification added (Illustra GenomicPrep Kit; GE Healthcare). Successful extraction was measured by PCR of the partial fragments of an ITS-2 gene and 16S rRNA gene in a previous study (Duncan et al. 2021); there were no failed DNA extraction or notable PCR inhibitor in this study. *Rickettsia* spp. was detected and identified using previously described and optimized 17-kDa and ompA gene targets (Sumner et al. 2007, Heise et al. 2010). An *E. canis-*specific PCR targeting a 16S rRNA gene fragment was performed to determine if *E. canis* was present in the subsample described in the previous paragraph (Chen et al. 1994, Dawson et al. 1996). *Babesia* spp. primers targeting fragments of 18S and the large subunit region rRNA genes were used for PCR on the subset of ticks described above (Kim et al. 2013, Qurollo et al. 2017). All amplified products were confirmed on a 2% agarose gel, column purified, and sequenced with an ABI 3730 capillary sequencer (Applied Biosystems, Foster City, CA, USA) at the Oklahoma State University Molecular Core Facility (Stillwater, Oklahoma, USA). High-quality electropherograms were verified by visual inspection and sequences were compared to those available in GenBank (https://blast.ncbi.nlm.nih.gov/Blast.cgi).

### Data analysis

Nucleic acid alignment and phylogenetic analyses for *Rickettsia* spp. were conducted using MacVector with Assembler 18.0.0 (MacVector, Inc.). Descriptive statistics (proportions and corresponding exact binomial 95% confidence intervals [CI]) were calculated alongside each reported prevalence with Microsoft Excel. Inferential statistics (chi-squares) were calculated with Microsoft Excel (Microsoft Office Professional Plus 2016) to compare *Rickettsia* spp. prevalence against tick stage, tick host, and tick U.S. region origin. The level of significance was set at *α* = 0.05, and Bonferroni correction was applied to multiple comparisons (*i.e.*, U.S. regions). Maps were developed with MapViewer 8.7.752 (Golden Software, LLC, Golden, CO, USA) with geographic regions (Northeast, South, Midwest, and West) as previously established (Blagburn et al. 1996).

## Results

**FIG. 1.**
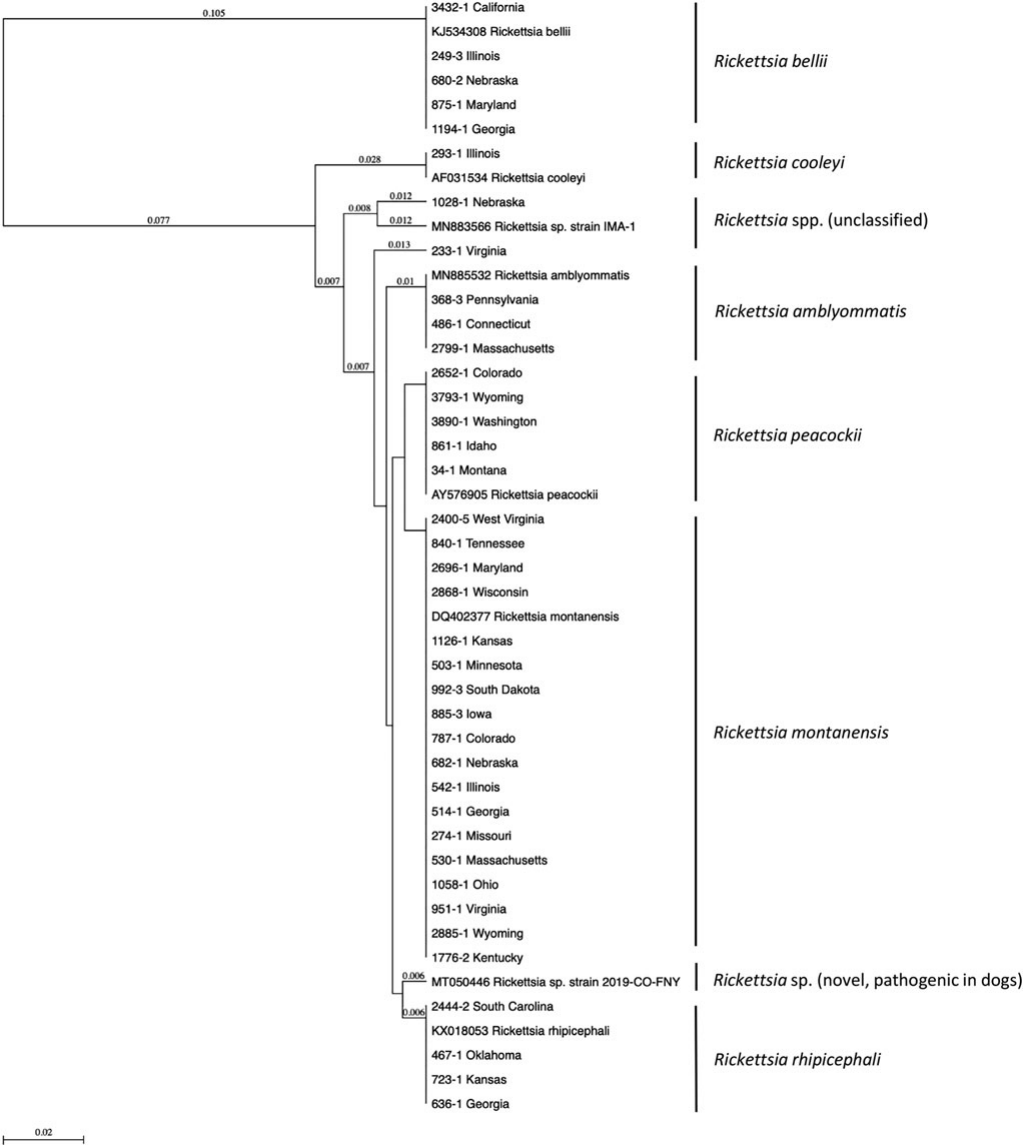
Phylogenetic relationship of representative *Rickettsia* spp. Seventeen kilodalton sequences detected in *Dermacentor* spp. from dogs and cats across the United States. Sequences are identified by tick accession number and state of geographic origin or by accession number of comparator sequences.

*Rickettsia* spp. was found in 12.5% (106/848; 95% CI 10.4–14.9) of the ticks tested with 11.9% (98/827; 95% CI 9.8–14.2) and 66.7% (8/12; 95% CI 38.8–86.5) of *D. variabilis* and *D. andersoni*, respectively, having detectable *Rickettsia* species; *Rickettsia* spp. was not detected in any *D. albipictus* (*n* = 9) (Table 1). Out of the 544 female ticks evaluated, 11.9% (65/544; 95% CI 9.5–15.0) had a *Rickettsia* sp. identified, and similarly, 13.8% (41/297; 95% CI 10.3–18.2) of the male ticks contained a *Rickettsia* species (chi-squared test, *χ*^2^ = 0.44, df = 1, *p* = 0.5). *Rickettsia* spp. was not detected in the immature ticks tested (*n* = 2 *D. variabilis* and *n* = 5 *D. albipictus*). Host (dog versus cat) did not significantly affect prevalence of *Rickettsia* species in this tick population (chi-squared test, *χ*^2^ = 0.01, df = 1, *p* = 0.9) as 12.5% (96/770; 95% CI 10.3–15.0) of ticks collected from dogs had a *Rickettsia* sp., while 12.8% (10/78; 95% CI 6.9–22.2) of ticks from cats had a *Rickettsia* sp. identified. *Rickettsia* spp. identified include *R. montanensis* (7.6%; 64/848; 95% CI 5.9–9.5), *R. bellii* (1.8%; 15/848; 95% CI 1.1–2.9), *Rickettsia rhipicephali* (1.5%; 13/848; 95% CI 0.9–2.6), *R. peacockii* (0.9%; 8/848; 95% CI 0.4–1.9), *R. amblyommatis* (0.4%; 3/848; 95% CI; 0.1–1.1), *R. cooleyi* (0.1%; 1/848; 95% CI 0.0–0.7), and two unclassified *Rickettsia* spp. (0.2%; 2/848; 95% CI 0.0–0.9) (Fig. 1). No tick had more than one *Rickettsia* spp. identified using both gene targets.

**FIG. 2.**
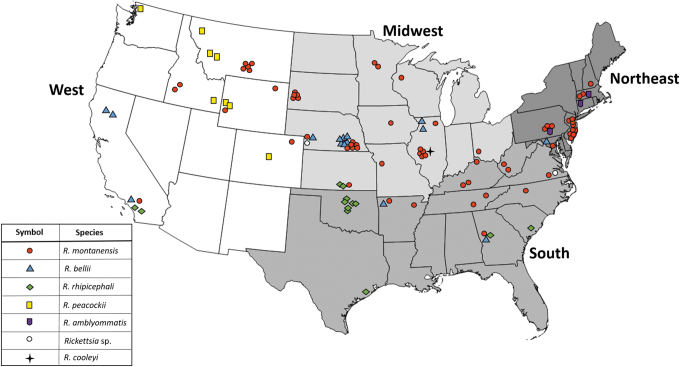
Geographic distribution of *Rickettsia* spp. detected in *Dermacentor* spp. from dogs and cats across the United States; each symbol represents a tick with a detectable *Rickettsia* spp. (*n* = 106). Color images are available online.

Overall prevalence of *Rickettsia* spp. in ticks from the four different defined regions (Northeast, South, Midwest, and West) was not significantly different (chi-squared test, *χ*^2^ = 7.06, df = 3, *p* = 0.07) with *Rickettsia* spp. detected in 13.4% (38/284; 95% CI 9.9–17.9) of Midwest ticks, 13.0% (24/185; 95% CI 8.8–18.6) of ticks from the West, 11.8% (24/203; 95% CI 8.0–17.0) of ticks from the South, and 11.0% (20/181; 95% CI 7.2–16.5) of ticks from the Northeast (Table 2). Across every region, *R. montanensis* was most often detected in the evaluated ticks. In addition, *R. bellii* was detected in ticks originating from every region. *Rickettsia peacockii* was only detected in *D. andersoni* from the Rocky Mountain states in the West, and no other *Rickettsia* spp. was present in the evaluated *D. andersoni* (*n* = 12). *R. rhipicephali* was detected in ticks that originated solely in the southern portions of the United States, and the three ticks with *R. amblyommatis* were submitted from states in the Northeast (Fig. 2).

**Table 2.** Prevalence, and Corresponding 95% Confidence Intervals, of *Rickettsia* Species in Adult *Dermacentor* spp. Collected from Dogs and Cats in Different Regions of the United States, February 2018 to November 2019

Rickettsia spp.	Northeast	Midwest	South	West	
	Dermacentor variabilis	D. variabilis	D. variabilis	D. variabilis	Dermacentor andersoni
*R. amblyommatis* ^a^	3/181 (1.7%) (0.3–5.0)	0 (0.0–1.6)	0 (0.0–2.2)	0 (0.0–2.6)	0
*R. bellii*	1/181 (0.6%) (0.0–3.4)	9/284 (3.2%) (1.6–6.0)	2/203 (1.0%) (0.0–3.8)	3/173 (1.7%) (0.4–5.2)	0
*R. cooleyi*	0 (0.0–2.5)	1/284 (0.4%) (0.0–2.2)	0 (0.0–2.2)	0 (0.0–2.6)	0
*R. montanensis*	16/181 (8.8%) (5.4–14.0)	25/284 (8.8%) (6.0–12.7)	12/203 (5.9%) (3.3–10.1)	11/173 (6.4%) (3.5–11.1)	0
*R. peacockii*	0 (0.0–2.5)	0 (0.0–1.6)	0 (0.0–2.2)	0 (0.0–2.6)	8/12 (66.7%) (38.8–86.5)
*R. rhipicephali* ^b^	0 (0.0–2.5)	2/284 (0.7%) (0.0–2.7)	9/203 (4.4%) (2.2–8.3)	2/173 (1.2%) (0.1–4.4)	0
Unclassified *Rickettsia* sp.	0 (0.0–2.5)	1/284 (0.4%) (0.0–2.2)	1/203 (0.5%) (0.0–3.0)	0 (0.0–2.6)	0
Total	20/181 (11.0%) (7.2–16.5)	38/284 (13.4%) (9.9–17.9)	24/203 (11.8%) (8.0–17.0)	16/173 (9.2%) (5.7–14.6)	8/12 (66.7%) (38.8–86.5)

*Dermacentor andersoni* (*n* = 12) was only submitted from the western United States; *Rickettsia* spp. was not detected in any *Dermacentor albipictus* (*n* = 9).

^a^ Dogs with *Dermacentor variabilis* in which *Rickettsia amblyommatis* was identified were co-infested with *Ixodes scapularis* (*n* = 1) or not co-infested with other tick species at the time of presentation (*n* = 2).

^b^ Dogs with *D. variabilis* in which *Rickettsia rhipicephali* was identified were co-infested with *Amblyomma americanum* (*n* = 3), *Amblyomma maculatum* (*n* = 2), *I. scapularis* (*n* = 1), and *Rhipicephalus sanguineus sensu* lato (*n* = 1), or were not co-infested with other tick species at the time of presentation (*n* = 8).

*E. canis* was not identified in any of the 150 *Dermacentor* spp. from 102 dogs (123 ticks) and 21 cats (27 ticks) from 17 states tested. A subset of *Dermacentor* spp. (*n* = 189) from 132 dogs (157 ticks) and 26 cats (32 ticks) from 20 states was tested for *Babesia* spp., and two nymphal *D. albipictus* had detectable *Babesia conradae* (1.1%; 2/189; 95% CI 0.0–4.0). The ticks originated from Minnesota and Colorado in October and November, respectively, and were both collected from adult cats.

## Discussion

Even though *D. variabilis* and *D. andersoni* are considered primary vectors of *R. rickettsii*, the causative agent of RMSF, we did not detect this pathogen in any *Dermacentor* tick (*n* = 848) collected from dogs and cats in 44 U.S. states. Instead, we identified various other SFGR, which support data from other studies reporting a range of SFGR and a low prevalence of *R. rickettsii* in *Dermacentor* ticks (Moncayo et al. 2010, Fritzen et al. 2011, Trout Fryxell et al. 2015, Santanello et al. 2018, Dykstra et al. 2020, Francis et al. 2020, Luedtke et al. 2020, Occi et al. 2020). In fact, when identified in *D. variabilis*, *R. rickettsii* has a reported prevalence less than 1%; however, this is in contrast to previous reports of greater than 8% of ticks harboring *R. rickettsii* (Schriefer and Azad 1994, Stromdahl et al. 2011, Kakumanu et al. 2018, Hecht et al. 2019). This change to a lack, or rarity, of *R. rickettsii* in its primary tick vectors could be explained by evidence suggesting tick endosymbionts have the potential to alter tick physiology and transmission of pathogens as indicated by the inverse relationship between *R. rickettsii* and *R. peacockii* in *D. andersoni* and documentation of *R. peacockii* infection, excluding *R. rickettsii* infection in the same tick (Burgdorfer et al. 1981, Narasimhan and Fikrig 2015). Approximately two-thirds (66.7%) of *D. andersoni* had *R. peacockii* identified in this study, although only 12 specimens were evaluated. Other studies have documented *R. peacockii* in 1.3–80% of *D. andersoni* tested and found no *R. rickettsii* in those specimens (Burgdorfer et al. 1981, Niebylski et al. 1997, Francis et al. 2020, Lefcort et al. 2020).

For *D. variabilis*, a similar mechanism may exist as data suggest this tick is unable to maintain two different *Rickettsia* spp. through transovarial transmission (Macaluso et al. 2002). In addition, other tick genera may be responsible for transmitting the causative agent of RMSF more frequently than originally recognized. In the southwestern United States, the brown dog tick, *R. sanguineus* s.l., is capable of transmitting *R. rickettsii*, while *Haemaphysalis longicornis* and *A. americanum* can successfully maintain and transmit *R. rickettsii* under laboratory conditions*,* although their transmission potential in nature is not fully known (Demma et al. 2005, Labruna et al. 2008, Levin et al. 2017, Stanley et al. 2020). Besides *R. rickettsii*, other SFGR can cause significant disease in humans and therefore may be contributing to the rise in reported cases of rickettsiosis since many SFGR cross-react on commonly used serologic assays (Moncayo et al. 2010). Two such examples include *R. philipii* (*Rickettsia* 364D), the causative agent of Pacific Coast tick fever, transmitted by *D. occidentalis* along its range in California, and *R. parkeri* transmitted by *A. maculatum* in the southeastern United States (Paddock et al. 2010, 2018, Stromdahl et al. 2011, Yoshimizu and Billeter 2018).

In this study, *R. montanensis* was the most prevalent *Rickettsia* spp. identified in *D. variabilis,* with this agent detected in over half (60.4%; 64/106; 95% CI 50.4–69.8) of the *Rickettsia*-positive ticks. Geographically, *R. montanensis* was found in ticks collected from every U.S. region and in 26 of the 44 states in which ticks were evaluated. Regional surveys from 22 states across the United States similarly found *R. montanensis* in *D. variabilis* (Table 1). Although the pathogenicity of *R. montanensis* in dogs and cats is currently unknown, mild disease has been induced in laboratory-reared guinea pigs and the agent was identified in a *D. variabilis* collected from a human before the development of a rash illness (McQuiston et al. 2012, Snellgrove et al. 2021). When considering the case report of human disease attributed to this SFGR, the widespread distribution of *R. montanensis* reported in this study is noteworthy. Before the human case report, *R. montanensis* was considered solely a nonpathogenic symbiont of *D. variabilis* (McQuiston et al. 2012, Parola et al. 2013). In ticks, the transition from endosymbiont to pathogen is not novel as evidenced by the emergence of disease-causing strains of *Coxiella* following numerous mutations and acquisition of new virulence factors (Duron et al. 2015). A recent example comes from the southern United States where a novel *Rickettsia* sp. has been identified as a cause of rickettsiosis in dogs (Wilson et al. 2020). Further investigation of intracellular pathogens and their evolution suggests this transition to a medically relevant pathogen may be achieved through inaccurate replication of the nonpathogenic *Rickettsia* spp. widely prevalent in ticks (Darby et al. 2007, Bonnet et al. 2017).

In total, eight different *Rickettsi*a spp. were identified in this study. Diversity of SFGR appears common in surveys of *D. variabilis* with as many as 10 species having been found within a population of this tick (Stromdahl et al. 2011, Trout Fryxell et al. 2015, Kakumanu et al. 2018, Hecht et al. 2019). In general, *Rickettsia* spp. makes up the largest proportion of microbial biomass of *D. variabilis* (Sanchez-Vicente et al. 2019). Although none of the ticks examined in this study had more than one *Rickettsia* spp. detected, it has been reported that multiple *Rickettsia* spp. can infect a single *D. variabilis* or *D. andersoni*; however, the transovarial transmission of more than one *Rickettsia* species may not occur (Macaluso et al. 2002, Carmichael and Fuerst 2010, Kakumanu et al. 2018, Francis et al. 2020). Regardless, detecting the presence of *Rickettsia* spp. or any other tick-borne pathogen in a given tick does not confirm that species plays a role in transmission; vector competence can only be assessed through experimental transmission studies.

*Dermacentor* spp. is most well known for their ability to transmit *Rickettsia* spp., but this genus may also serve as a vector for other medically relevant tick-borne pathogens. For instance, two *D. variabilis* ticks from Mexico, an endemic region for canine ehrlichiosis, were found to have *E. canis*, and experimentally, *D. variabilis* is capable of transmitting *E. canis* (Johnson et al. 1998, Sosa-Gutierrez et al. 2016). However, none of the *Dermacentor* tested (*n* = 150) in this study had detectable *E. canis*. The ticks in this study were submitted primarily from regions of the United States where canine infection with *E. canis* is relatively rare, which may have precluded detection of the organism (Beall et al. 2012). Moreover, as with detection alone, proving vector competence in an experimental study does not confirm relevance to transmission in nature. *Ixodes scapularis* ticks from Louisiana, a state in the southern United States, are capable of transmitting *Borrelia burgdorferi* in the laboratory, but, due to differences in tick phenology, host preferences, and questing behavior, do not sustain a maintenance system for this agent in the field (Jacobs et al. 2003, Goddard et al. 2015, Ginsberg et al. 2021).

In addition, some agents of babesiosis are suspected to be transmitted by *Dermacentor* spp. (Kjemtrup and Conrad 2006, Shock et al. 2014, Swei et al. 2019). In a multistate survey, 2.7% of the *D. variabilis* tested had detectable *Babesia* spp., and *D. albipictus* has been documented in the transmission cycle of *B. duncani* to humans (Shock et al. 2014, Swei et al. 2019). In this study, *B. conradae*, a potentially fatal agent infecting dogs in the southern United States, was identified in two *D. albipictus* collected from cats. The majority of reported cases of *B. conradae* is associated with kennels of coyote-hunting dogs, and although ticks are suspected to play a role in transmission, to date, no tick has been named as the primary vector (Kjemtrup and Conrad 2006, Dear et al. 2018). Our findings suggest further work is needed to elucidate the relationship, if any, between *D. albipictus* and *B. conradae*.

This study had several limitations. The ticks examined in this study were collected from pets and may have ingested a blood meal before submission; therefore, the results of this study do not implicate a particular *Dermacentor* sp. as the primary vector for the pathogens identified. For instance, *R. rhipicephali* is more often identified in *R. sanguineus* (s.l.), *R. amblyommatis* is associated frequently with *A. americanum*, and *R. cooleyi* with *Ixodes* spp., and yet these agents have been found in various tick species (Hayes and Burgdorfer 1979, Moncayo et al. 2010, Fritzen et al. 2011, Barrett et al. 2014). In addition, if multiple *Rickettsia* spp. were present in a tick, reaction conditions may have favored amplification of the target in greater abundance or failed to amplify some *Rickettsia* spp. at all, precluding their detection. Similarly, although ticks were available from 44/50 states for analysis, our reliance on passive surveillance may have resulted in inadequate numbers of tick specimens to test from a given state, leading to failure of detection of less common pathogens.

## Conclusion

This study highlights the diversity of SFGR in *D. variabilis* collected from dogs and cats across the United States. The majority of the detected species currently has an unknown pathogenicity in pets, but may induce cross-reacting antibodies on IFA, confounding diagnostic tests. Continued surveillance of tick-borne agents for the sake of veterinary and human health is warranted.
